# Circulating Fatty Acids and Prostate Cancer Risk: Individual Participant Meta-Analysis of Prospective Studies

**DOI:** 10.1093/jnci/dju240

**Published:** 2014-09-04

**Authors:** Francesca L. Crowe, Paul N. Appleby, Ruth C. Travis, Matt Barnett, Theodore M. Brasky, H. Bas Bueno-de-Mesquita, Veronique Chajes, Jorge E. Chavarro, Maria-Dolores Chirlaque, Dallas R. English, Robert A. Gibson, Graham G. Giles, Gary E. Goodman, Susanne M. Henning, Rudolf Kaaks, Irena B. King, Lawrence N. Kolonel, Alan R. Kristal, Marian L. Neuhouser, Song-Yi Park, Gianluca Severi, Afshan Siddiq, Meir J. Stampfer, Pär Stattin, Catherine M. Tangen, Anne Tjønneland, Dimitrios Trichopoulos, Rosario Tumino, Lynne R. Wilkens, Timothy J. Key, Naomi E. Allen, on behalf of the Endogenous Hormones, Nutritional Biomarkers and Prostate Cancer Collaborative Group

**Affiliations:** **Affiliations of authors**: Cancer Epidemiology Unit, Nuffield Department of Population Health, University of Oxford, Oxford, UK (FLC, PNA, RCT, TJK); Division of Public Health Sciences, Fred Hutchinson Cancer Research Center, Seattle, WA (MB, GEG, IBK, MLN); Department of Internal Medicine, Division of Cancer Prevention and Control, The Ohio State University College of Medicine, Columbus, OH (TMB); National Institute for Public Health and the Environment – RIVM, Bilthoven, the Netherlands (HBB); Department of Gastroenterology and Hepatology, University Medical Centre, Utrecht, the Netherlands (HBB); School of Public Health, Imperial College London, London, UK (HBB); Nutrition and Metabolism Section and the Nutritional Epidemiology Group, International Agency for Research on Cancer, Lyon, France (VC); Departments of Nutrition and Epidemiology, Harvard School of Public Health and Channing Division of Network Medicine, Boston, MA (JEC, MJS); Department of Medicine, Brigham and Women’s Hospital and Harvard Medical School, Boston, MA (JEC, MJS); Department of Epidemiology, Murcia Regional Health Authority, Spain (MC); CIBER Epidemiology and Public Health CIBERESP, Spain (MC); Cancer Epidemiology Centre, Cancer Council Victoria, Carlton, Victoria, Australia (DRE, GGG, GS); Centre for Molecular, Environmental, Genetic and Analytic Epidemiology, School of Population Health, The University of Melbourne, Victoria, Australia (DRE, GGG, GS); FOODplus Research Centre, Waite Campus, The University of Adelaide, Glen Osmond, South Australia, Australia (RAG); Center for Human Nutrition, David Geffen School of Medicine, University of California, Los Angeles, CA (SMH); Department of Cancer Epidemiology, German Cancer Research Center – DKFZ, Heidelberg, Germany (RK); Department of Internal Medicine, University of New Mexico, Albuquerque, NM (IBK); Cancer Epidemiology Program, University of Hawaii Cancer Center, Honolulu, HI (LNK, SYP, LRW); Cancer Prevention Program, Division of Public Health Sciences, Fred Hutchinson Cancer Research Center, Seattle, WA (ARK, CMT); Department of Genomics of Common Disease, School of Public Health, Imperial College London, London, UK (AS); Department of Surgery and Perioperative Sciences, Urology and Andrology, Umeå University, Umeå, Sweden (PS); Diet, Genes and Environment, Danish Cancer Society Research Center, Copenhagen, Denmark (AT); Department of Epidemiology, Harvard School of Public Health, Boston, MA (DT); Bureau of Epidemiologic Research, Academy of Athens, Athens, Greece, (DT); Hellenic Health Foundation, Athens, Greece (DT); Cancer Registry and Histopathology Unit, Civic and M. P. Arezzo Hospital, ASP Ragusa, Italy (RT); Clinical Trial Service Unit and Epidemiological Studies Unit, Nuffield Department of Population Health, University of Oxford, Oxford, UK (NEA).

## Abstract

**Background:**

Individual studies have suggested that some circulating fatty acids are associated with prostate cancer risk, but have not been large enough to provide precise estimates of associations, particularly by stage and grade of disease.

**Methods:**

Principal investigators of prospective studies on circulating fatty acids and prostate cancer were invited to collaborate. Investigators provided individual participant data on circulating fatty acids (weight percent) and other characteristics of prostate cancer cases and controls. Prostate cancer risk by study-specific fifths of 14 fatty acids was estimated using multivariable-adjusted conditional logistic regression. All statistical tests were two-sided.

**Results:**

Five thousand and ninety-eight case patients and 6649 control patients from seven studies with an average follow-up of 5.1 (SD = 3.3) years were included. Stearic acid (18:0) was inversely associated with total prostate cancer (odds ratio [OR] Q5 vs Q1 = 0.88, 95% confidence interval [CI] = 0.78 to 1.00, *P*_trend_ = .043). Prostate cancer risk was, respectively, 14% and 16% greater in the highest fifth of eicosapentaenoic acid (20:5n-3) (OR = 1.14, 95% CI = 1.01 to 1.29, *P*_trend_ = .001) and docosapentaenoic acid (22:5n-3) (OR = 1.16, 95% CI = 1.02 to 1.33, *P*_trend_ = .003), but in each case there was heterogeneity between studies (*P* = .022 and *P* < .001, respectively). There was heterogeneity in the association between docosapentaenoic acid and prostate cancer by grade of disease (*P* = .006); the association was statistically significant for low-grade disease but not high-grade disease. The remaining 11 fatty acids were not statistically associated with total prostate cancer risk.

**Conclusion:**

There was no strong evidence that circulating fatty acids are important predictors of prostate cancer risk. It is not clear whether the modest associations of stearic, eicosapentaenoic, and docosapentaenoic acid are causal.

International comparison studies have suggested that factors related to a western lifestyle, such as diet, may be important determinants of the worldwide variation in prostate cancer rates (1,2). A higher intake of foods such as red meat and dairy products, which are rich in saturated fatty acids, and a lower intake of marine-derived n-3 long-chain polyunsaturated fatty acids (eicosapentaenoic [20:5n-3] and docosahexaenoic acid [22:6n-3]) are dietary factors that have been proposed to increase the risk of prostate cancer (3). Because it is difficult to obtain accurate and precise information on the consumption of individual fatty acids using conventional assessment methods such as dietary questionnaires, circulating concentrations of fatty acids (in serum, plasma, erythrocyte membranes, or whole blood) may be a better measure for assessing the associations of individual fatty acids with prostate cancer risk (4,5). Furthermore, fatty acids in the blood also reflect the endogenous synthesis of certain fatty acids (5), a process that cannot be captured by assessing the dietary intake of fatty acids.

To date, the association between circulating fatty acids and risk of prostate cancer has been examined in 10 individual prospective studies (6–15), but these have not been large enough to provide precise estimates of associations, especially by stage and grade of disease. Moreover, it is difficult to evaluate the evidence for an association of some individual fatty acids with prostate cancer risk from the published data alone, because some studies have not presented data for the full range of fatty acids measured.

The Endogenous Hormones, Nutritional Biomarkers and Prostate Cancer Collaborative Group was established with the aim of reanalyzing individual data from prospective studies of the associations of circulating concentrations of hormones and nutritional biomarkers with risk of prostate cancer (16). The objective of this study was to examine the associations between the proportional concentrations of 14 circulating fatty acids and subsequent risk of prostate cancer in a collaborative reanalysis of individual participant data from seven prospective studies and to determine whether any associations differ by stage and grade of the disease or other factors.

## Methods

### Data Sources and Searches

Studies were eligible to join this collaborative group if they had data on circulating fatty acids measured in blood samples collected before the diagnosis of prostate cancer and had identified at least 75 incident cases during follow-up. Studies were identified through searches using the search terms “fatty acids” and “prostate cancer” on computerized bibliographic systems, including PubMed, Web of Science, Cochrane Library, and CancerLit, through the reference lists of publications identified in this search and through discussions with colleagues. In 2004, the principal investigators who had published studies on prostate cancer risk and endogenous sex hormones and growth factors measured in blood samples collected before the diagnosis of prostate cancer and which included at least 50 cases were invited to join the collaboration. In 2010, collaborators were invited to update their data on endogenous sex hormones and growth factors and to include any data on nutritional biomarkers and prostate cancer. Seven out of the nine published prospective studies that met the inclusion criteria provided fatty acid data for this collaboration (9–15). Two published studies of 141 case patients and 282 control patients (6) and 198 case patients and 198 control patients (7) invited to participate were unable to join this collaboration.

### Study Selection and Data Extraction

Individual participant data on circulating fatty acids were available from seven prospective studies: the Carotene and Retinol Efficacy Trial (CARET) (15), the European Prospective Investigation into Cancer and Nutrition (EPIC) study (10), the Melbourne Collaborative Cohort Study (MCCS) (13), the Multiethnic Cohort (MEC) (11), the Prostate Cancer Prevention Trial (PCPT) (12), the Physicians’ Health Study (PHS) (9), and the Selenium and Vitamin E Cancer Prevention Trial (SELECT) (14). These included 5098 case patients and 6649 control patients, representing almost 95% of the worldwide prospective data on fatty acids and prostate cancer. Where available, collaborators provided data for case patients and control patients on date, age, time of blood collection, fasting status, marital status, ethnicity, educational attainment, family history of prostate cancer, height, weight, smoking status, alcohol intake, and prostate-specific antigen (PSA) concentration. Information on stage and grade for prostate cancer case patients was also provided where available. The data obtained from the collaborators was summarized and then sent back to collaborators for final checking and confirmation. In order to provide a common definition across studies, a cancer was defined as being advanced if it was tumor-node-metastasis (TNM) stage T3 or T4 and/or N1+ and/or M1, stage III–IV, or the equivalent (that is, a tumor extending beyond the prostate capsule and/or lymph node involvement and/or distant metastases), localized if it was TNM stage T0 or T1 or T2 with no reported lymph node involvement or metastases, stage 0–II, or the equivalent (that is, a tumor which does not extend beyond the prostate capsule), or stage unknown. Aggressive disease was categorized as “yes” for TNM stage T4 and/or N1+ and/or M1 and/or stage IV disease or death from prostate cancer, “no” for TNM stage T0, T1, T2, or T3 with no reported lymph node involvement or metastases or the equivalent, or unknown. Prostate cancer was defined as high grade if the Gleason sum was at least 8 or the equivalent (undifferentiated), low grade if the Gleason sum was less than 8 or the equivalent (extent of differentiation good, moderate, or poor), or grade unknown. Grade was based on Gleason sums for CARET, PCPT, and SELECT (17), on extent of differentiation for MEC, and on a mixture of both systems for EPIC, MCCS, and PHS. It should be noted that in the previous publications from these studies a Gleason sum of 7 or above was classed as high grade, with the exception of PCPT, where a Gleason sum of 8 or above was used.

Supplementary Table 1 (available online) shows the characteristics of the seven studies included in this collaboration. Details of the recruitment of participants, informed consent, ethical approval, and inclusion criteria are available in the original publications (9–15). Two of the seven studies used a matched case-control study design nested within a prospective study (10,11). Three studies used a matched case-control study design nested within a randomized controlled trial (9,12,15). For this collaboration, the PCPT study contributed men in the placebo arm of the trial, because the intervention group (who were given the drug finasteride) had a lower risk of prostate cancer than the controls, but CARET and PHS included all men regardless of whether they were in the control or the intervention group. In these five studies (CARET, EPIC, MEC, PCPT, and PHS), blood samples were collected from men who were healthy at recruitment and then followed to identify those who developed prostate cancer. The fatty acid analyses were performed on blood samples from the participants diagnosed with incident prostate cancer and from the control participants who were matched to these cases on certain characteristics such as age at blood collection and date of blood collection (Supplementary Table 1, available online). One study (13) used a case-cohort design nested within a prospective study where fatty acids were measured for all incident cases of prostate cancer and a random sample of the cohort. For this analysis, matched case-control sets were created from the case-cohort study by randomly matching up to two control participants to each case by age at blood collection, date of recruitment, assay batch number, and country of birth. The SELECT study used a case-cohort design nested within a randomized controlled trial (14). For this analysis, one control was matched to each case by age at blood collection and ethnicity.

Details of the assay methods for the measurement of circulating fatty acids are shown in Supplementary Table 2 (available online); five of the studies measured the fatty acid composition of plasma or serum phospholipids, MEC measured fatty acids in erythrocyte membranes, and PHS measured the fatty acid composition of whole blood. Of the five studies that measured the fatty acid composition of serum or plasma phospholipids, four studies (CARET, MCCS, PCPT, and SELECT) used thin-layer chromatography to separate out the phospholipids, whereas the EPIC study used solid phase extraction.

### Data Synthesis and Analysis

For each of the 14 fatty acids, men were categorized into fifths of its circulating proportion (calculated as a relative weight percent of the total fatty acids), with cut points defined by the study-specific quintiles of the distribution within control participants. This was to allow for any systematic differences between the studies in assay methods and blood sample types (18). We also examined the associations of study-specific fifths of the following groups of fatty acids with prostate cancer risk: total n-6 polyunsaturated fatty acids (sum of linoleic, dihomo-gamma-linolenic, and arachidonic acid), total n-3 polyunsaturated fatty acids (sum of alpha-linolenic, eicosapentaenoic, docosapentaenoic, and docosahexaenoic acid), the ratio of total n-6 to n-3 polyunsaturated fatty acids, long-chain n-6 polyunsaturated fatty acids (sum of dihomo-gamma-linolenic and arachidonic acid), long-chain n-3 polyunsaturated fatty acids (sum of eicosapentaenoic, docosapentaenoic, and docosahexaenoic acid), and the ratio of long-chain n-6 to n-3 polyunsaturated fatty acids.

The main method of analysis was logistic regression conditioned on the matching variables within each study. To provide a summary measure of the odds ratio (for subgroup analyses) and to calculate a *P*_trend_, the categorical variable representing the fifths of the circulating fatty acid was replaced with a continuous variable that was scored as 0, 0.25, 0.5, 0.75, and 1; because the mid-points of the lowest and highest fifths are the 10^th^ and 90^th^ percentiles of the study-specific proportion of fatty acids, a unit increase in this variable can be taken to represent an 80 percentile increase in the study-specific proportion of the fatty acid. Body mass index (BMI; <25, 25–27.4, 27.5–29.9, ≥30kg/m^2^, or not known), height (<170, 171–175, 176–180, >180cm, or not known), marital status (married or cohabiting, not married and cohabiting, or not known), educational status (did not graduate from high school/secondary school/college, high school/secondary school/college graduates, university graduates, or not known), cigarette smoking (never smoker, past smoker, current smokers, or not known), and age at blood collection (continuous) were all statistically significantly related to prostate cancer risk in these analyses (*P* < .05) and were included in the conditional logistic regression models. For each fatty acid, heterogeneity in linear trends between studies was tested by comparing the χ^2^ values for models with and without an (study) x (linear trend) interaction term. To test whether the linear-trend odds ratio estimates for each fatty acid varied according to certain case-participant characteristics, odds ratios were estimated within a series of subsets for the following characteristics: age at diagnosis (<60, 60–69, or ≥70 years), years from blood collection to diagnosis (<3, 3–6, or ≥7 years), year of diagnosis (pre-1990, 1990–1994, 1995–1999, or 2000 onwards), stage of disease (localized or advanced), aggressive disease (no or yes), and grade of disease (low or high). Control patients in each matched set were assigned the value of their matched case for the case-defined factors (eg, age at diagnosis and years from blood collection to diagnosis). For PCPT where the matched sets contained multiple case patients, the proportion of case patients in each category (excluding the “unknowns”) of the case-defined variable within the matched set was calculated, and the controls in the matched set were randomly allocated to these categories in the same proportion. For aggressive disease, we also conducted further analyses restricted to these case patients and their matched control patients, calculating odds ratios in fifths of the distribution in control patients. Subgroup analyses were also conducted according to the following participant characteristics: age at blood draw (<60 or ≥60 years), PSA at blood draw (<2 or ≥2ng/mL, based on the median value), university or higher education (no or yes), BMI (<25 or ≥25kg/m^2^), cigarette smoking (never/past smoker or current smoker), usual alcohol consumption (<10 or ≥10g/day), and family history of prostate cancer (no or yes). Tests for heterogeneity for the case-defined factors were obtained by fitting separate models for each subgroup and assuming independence of the odds ratios using a method analogous to a meta-analysis. Tests for heterogeneity for the non-case-defined factors were assessed using a χ^2^-test of interaction between subgroup and the continuous trend test variable. Results in the figures are presented as squares and lines, representing the odds ratios and corresponding 95% confidence intervals (CIs), respectively. The position of the square indicates the value of the odds ratio, while the size of the square is inversely proportional to the variance of the logarithm of the odds ratio and indicates the amount of statistical information available for that particular estimate. The open diamonds (the lateral points of which are the 95% CIs) represent the overall odds ratio for an 80 percentile increase in the individual fatty acids.

All statistical analyses were carried out using Stata (StataCorp, 2011, *Stata Statistical Software: Release 13*, College Station, TX). All *P* values reported are two-sided and a *P* value less than .05 was considered statistically significant.

## Results

Five thousand and ninety-eight case patients and 6649 control patients from seven studies with an average follow-up of 5.1 (SD 3.3) years were included. The characteristics of participants in each study according to their case-control status are shown in Table 1. The mean age at recruitment across studies ranged from 58 to 69 years, and the average BMI of men ranged from 24 to 29kg/m^2^. The majority of men were married (or cohabiting), and the proportion of men who were current smokers ranged from less than 10% in PCPT, PHS, and SELECT to over 50% in the CARET study. The mean alcohol intake ranged from less than 10g/day in PCPT, PHS, and SELECT to over 20g/day in EPIC and MEC.

**Table 1.** Participant characteristics by study and case-control status^*^

Study	Case-control status	Number of participants	Mean (SD) age at recruitment, y	Mean (SD) BMI, kg/m^2^	Married or cohabiting, %	Higher education, %	Current smokers, %	Mean (SD) intake of alcohol, g/d
CARET	Case patients	666	60.0 (5.7)	28.2 (4.3)	81.7	28.1	51.7	17.6 (25.3)
	Control patients	1282	59.7 (5.8)	28.2 (4.4)	81.7	23.9	52.3	15.6 (26.0)
EPIC	Case patients	964	59.9 (5.8)	26.6 (3.4)	88.8	27.0	24.4	21.6 (24.8)
	Control patients	1063	59.6 (5.7)	26.9 (3.6)	88.8	22.7	29.9	21.2 (24.4)
MCCS	Case patients	549	60.9 (6.4)	27.2 (3.5)	80.2	22.6	9.7	19.1 (24.4)
	Control patients	1019	58.4 (7.2)	27.2 (3.7)	81.2	22.5	13.2	21.1 (25.8)
MEC	Case patients	377	68.6 (7.1)	26.6 (4.1)	76.9	32.2	15.0	23.4 (42.8)
	Control patients	732	68.5 (7.2)	27.0 (4.2)	78.8	31.1	12.1	21.8 (38.6)
PCPT	Case patients	995	63.3 (5.5)	27.4 (4.1)	87.5	39.4	6.7	9.7 (16.1)
	Control patients	995	63.3 (5.5)	27.6 (4.0)	87.7	37.1	7.5	9.0 (13.9)
PHS	Case patients	488	58.2 (7.8)	24.7 (2.5)	N/A	100.0	7.6	7.3 (6.1)
	Control patients	499	58.1 (7.6)	24.4 (2.5)	N/A	100.0	7.4	7.0 (6.1)
SELECT	Case patients	1059	63.8 (6.1)	28.6 (4.4)	83.5	56.0	5.1	9.2 (15.3)
	Control patients	1059	63.9 (6.6)	28.7 (4.7)	83.2	52.1	7.4	9.1 (15.0)

^*^ The numbers of case patients and control patients are the maximum numbers for whom fatty acid measurements were available, and numbers varied by fatty acid. BMI = body mass index; CARET = Carotene and Retinol Efficacy Trial; EPIC = European Prospective Investigation into Cancer and Nutrition; MCCS = Melbourne Collaborative Cohort Study; MEC = Multiethnic Cohort; N/A = data not available for this study; PCPT = Prostate Cancer Prevention Trial; PHS = Physicians’ Health Study; SELECT Selenium and Vitamin E Cancer Prevention Trial.

Most of the case patients were older than 60 years at diagnosis and were diagnosed with prostate cancer from 1995 onwards (Table 2). The proportion of case patients whose blood was collected at least seven years before diagnosis ranged from approximately 1% in SELECT to 74% in PHS. Of the case patients with information on stage of disease, the majority were localized, ranging from 71% in EPIC to 99% in SELECT. Similarly, the proportion of case patients with low-grade (that is, a Gleason score <8 or equivalent) ranged from 86% in EPIC to 100% in MEC.

**Table 2.** Characteristics of the case participants with prostate cancer by study

Study	Age at diagnosis, %			Date of diagnosis, %			Years from blood collection to diagnosis, %			Stage of disease, %*			Aggressive disease, %*			Grade of disease, %†		
	<60 y	60–69 y	≥70 y	Pre-1990	1990–94	1995 onwards	<3 y	3–6 y	≥7 y	Localized	Advanced	n/k	No	Yes	n/k	Low	High	n/k
CARET	11.3	55.3	33.5	2.7	29.4	67.9	19.4	35.1	45.5	66.5	20.0	13.5	76.7	10.7	12.6	81.7	11.3	7.1
EPIC	19.2	65.4	15.5	0	0.5	99.5	30.6	56.2	13.2	52.1	21.2	26.8	53.8	24.9	21.3	66.5	10.8	22.7
MCCS	12.4	54.3	33.3	0	15.1	84.9	26.0	35.7	38.3	89.4	9.1	1.5	96.7	1.8	1.5	84.3	13.7	2.0
MEC	7.4	36.3	56.2	0	0	100	79.6	17.8	2.7	0	0	100	0	4.8	95.2	95.2	0	4.8
PCPT	1.4	49.9	48.6	0	0.1	99.9	9.0	27.5	63.4	96.0	1.5	2.5	96.7	0.8	2.5	93.2	4.6	2.2
PHS	14.1	50.0	35.9	26.8	58.6	14.5	7.6	18.4	74.0	81.4	14.8	3.9	76.4	20.3	3.3	88.5	8.2	3.3
SELECT	12.3	54.9	32.9	0	0	100	55.6	43.2	1.2	97.5	0.9	1.5	97.4	1.1	1.5	84.3	7.6	8.1

^*^ Stage of disease was defined as being advanced if it was tumor-node-metastasis (TNM) stage T3 or T4 and/or N1+ and/or M1, stage III–IV, or approximate equivalent (that is, a tumor extending beyond the prostate capsule and/or lymph node involvement and/or distant metastases), localized if it was TNM stage T0 or T1 or T2 with no reported lymph node involvement or metastases, stage 0–II, or approximate equivalent (that is, a tumor that does not extend beyond the prostate capsule), or stage unknown (n = 90 in CARET, n = 258 in EPIC, n = 8 in MCCS, n = 377 in MEC, n = 25 in PCPT, n = 19 in PHS, and n = 16 in SELECT). Aggressive disease was categorized as “yes” for TNM stage T4 and/or N1+ and/or M1 and/or stage IV disease or death from prostate cancer, “no” for TNM stage T0, T1, T2, or T3 with no reported lymph node involvement or metastases or the equivalent, or unknown (n = 84 in CARET, n = 205 in EPIC, n = 8 in MCCS, n = 359 in MEC, n = 25 in PCPT, n = 16 in PHS, and n = 16 in SELECT).

^†^ Grade of disease defined as high grade if the Gleason sum was at least 8 or approximate equivalent (that is, extent of differentiation of “none”), low grade if the Gleason sum was less than 8 or approximate equivalent (that is, extent of differentiation of “poor,” “moderate,” or “good”) or grade unknown (n = 47 in CARET, n = 219 in EPIC, n = 11 in MCCS, n = 18 in MEC, n = 22 in PCPT, n = 16 in PHS, and n = 86 in SELECT).

The medians and interquartile ranges of the 14 fatty acids that make up approximately 90% to 95% of total fatty acids for case patients and control patients in each of the studies are shown in Table 3. The median proportions of the five fatty acids making up about 80% of the total fatty acids in the five studies (CARET, EPIC, MCCS, PCPT, and SELECT) that measured phospholipid fatty acids in serum or plasma were around 25% to 27% for palmitic acid (16:0), 11% to 14% for stearic acid (18:0), 8% to 10% for oleic acid (18:1n-9), 19% to 26% for linoleic acid (18:2n-6), and 9% to 11% for arachidonic acid (20:4n-6). For the fatty acid composition of erythrocyte membranes measured in MEC, these median values were 30%, 22%, 16%, 12%, and 9%, respectively, and, for the fatty acid composition of whole blood in the PHS, they were 20%, 10%, 17%, 25%, and 10%, respectively. There were no large differences in the median proportions of fatty acids between case patients and control patients.

**Table 3.** Proportion (weight %) of individual fatty acids by case-control status in each study*

Category	**CARET**		**EPIC**		**MCCS**		**MEC**		**PCPT**		**PHS**		**SELECT**	
	Case patients	Control patients	Case patients	Control patients	Case patients	Control patients	Case patients	Control patients	Case patients	Control patients	Case patients	Control patients	Case patients	Control patients
	n = 666†	n = 1282	n = 964	n = 1063	n = 549	n = 1019	n = 377	n = 732	n = 995	n = 995	n = 488	n = 499	n = 1059	n = 1059
**Fraction measured**	Serum phospholipids		Plasma phospholipids		Plasma phospholipids		Erythrocyte membranes		Serum phospholipids		Whole blood		Plasma phospholipids	
**Saturated fatty acids**														
Myristic acid (14:0)	0.24 (0.20–0.29)	0.24 (0.19–0.29)	0.31 (0.25–0.38)	0.31 (0.25–0.39)	0.23 (0.19–0.28)	0.23 (0.19–0.27)	–‡	–	0.26 (0.22–0.30)	0.26 (0.23–0.30)	0.44 (0.32–0.62)	0.41 (0.30–0.61)	0.26 (0.21–0.30)	0.26 (0.21–0.31)
Pentadecanoic acid (15:0)	0.13 (0.11–0.15)	0.13 (0.11–0.15)	0.16 (0.13–0.18)	0.16 (0.13–0.19)	0.17 (0.14–0.21)	0.17 (0.14–0.20)	–	–	0.14 (0.12–0.17)	0.14 (0.12–0.16)	0.14 (0.12–0.17)	0.14 (0.12–0.17)	0.14 (0.12–0.17)	0.14 (0.12–0.17)
Palmitic acid (16:0)	25.8 (24.7–27.0)	25.7 (24.7–26.8)	27.0 (25.4–28.5)	27.1 (25.4–28.6)	25.4 (24.5–26.4)	25.4 (24.4–26.4)	30.3 (29.3–31.4)	30.3 (29.3–31.1)	25.6 (24.6–26.5)	25.5 (24.8–26.5)	19.7 (18.7–20.9)	19.6 (18.5–20.7)	25.4 (24.3–26.4)	25.4 (24.4–26.5)
Heptadecanoic acid (17:0)	0.38 (0.32–0.43)	0.38 (0.33–0.44)	0.39 (0.34–0.44)	0.39 (0.34–0.44)	–	–	–	–	0.41 (0.36–0.44)	0.40 (0.36–0.44)	0.35 (0.32–0.38)	0.35 (0.31–0.38)	0.40 (0.36–0.45)	0.40 (0.36–0.44)
Stearic acid (18:0)	13.8 (13.1–14.6)	13.9 (13.2–14.6)	11.5 (10.9–12.1)	11.5 (10.9–12.1)	14.3 (13.7–15.1)	14.3 (13.6–15.1)	21.9 (20.8–22.8)	21.9 (20.9–23.0)	13.9 (13.2–14.5)	13.9 (13.2–14.5)	10.0 (9.4–10.4)	10.0 (9.5–10.5)	13.9 (13.2–14.6)	13.9 (13.2–14.6)
**Monounsaturated fatty acids**														
Palmitoleic acid (16:1n-7c)	0.38 (0.30–0.51)	0.37 (0.29–0.49)	0.78 (0.63–0.95)	0.79 (0.65–0.96)	0.36 (0.28–0.46)	0.37 (0.29–0.50)	0.39 (0.30–0.53)	0.39 (0.30–0.52)	0.39 (0.32–0.49)	0.39 (0.31–0.49)	1.02 (0.79–1.39)	0.95 (0.73–1.26)	0.37 (0.29–0.47)	0.37 (0.29–0.48)
Oleic acid (18:1n-9c)	7.7 (7.0–8.5)	7.6 (6.9–8.4)	10.1 (9.1–11.3)	10.3 (9.3–11.3)	9.3 (8.4–10.4)	9.5 (8.5–10.7)	16.1 (14.9–17.4)	16.1 (14.8–17.4)	7.5 (7.0–8.1)	7.6 (7.0–8.2)	16.9 (15.6–18.4)	16.5 (15.3–17.9)	7.6 (7.0–8.5)	7.6 (6.9–8.4)
**n-3 polyunsaturated fatty acids**														
α-linolenic acid (18:3n-3)	0.10 (0.09–0.11)	0.10 (0.09–0.12)	0.26 (0.18–0.33)	0.26 (0.19–0.34)	0.14 (0.11–0.19)	0.14 (0.10–0.20)	0.47 (0.32–0.69)	0.47 (0.34–0.68)	0.14 (0.12–0.17)	0.14 (0.12–0.17)	0.36 (0.29–0.45)	0.35 (0.29–0.44)	0.13 (0.11–0.17)	0.13 (0.11–0.17)
Eicosapentaenoic acid (20:5n-3)	0.57 (0.44–0.75)	0.56 (0.42–0.75)	1.24 (0.90–1.79)	1.20 (0.85–1.74)	0.98 (0.75–1.30)	0.94 (0.71–1.31)	0.56 (0.40–0.79)	0.53 (0.41–0.74)	0.58 (0.43–0.76)	0.55 (0.43–0.72)	1.74 (1.48–2.01)	1.79 (1.51–2.06)	0.62 (0.45–0.90)	0.58 (0.43–0.84)
n-3 docosapentaenoic acid (22:5n-3)	0.80 (0.71–0.91)	0.81 (0.71–0.91)	1.20 (1.03–1.38)	1.21 (1.02–1.39)	1.25 (1.08–1.42)	1.24 (1.08–1.41)	1.44 (1.20–1.86)	1.45 (1.21–1.80)	0.87 (0.77–0.99)	0.85 (0.74–0.96)	0.94 (0.84–1.06)	0.97 (0.86–1.09)	0.90 (0.79–1.02)	0.86 (0.75–0.99)
Docosahexaenoic acid (22:6n-3)	2.54 (2.11–3.14)	2.57 (2.10–3.15)	4.36 (3.63–5.25)	4.28 (3.51–5.08)	3.88 (3.25–4.57)	3.80 (3.24–4.48)	6.38 (5.32–7.59)	6.36 (5.30–7.59)	2.71 (2.28–3.35)	2.69 (2.20–3.25)	2.18 (1.79–2.61)	2.19 (1.80–2.78)	3.03 (2.39–3.76)	2.90 (2.31–3.63)
**n-6 polyunsaturated fatty acids**														
Linoleic acid (18:2n-6)	20.7 (18.8–22.4)	20.6 (18.9–22.3)	25.9 (23.4–28.2)	25.7 (23.2–28.1)	19.9 (18.1–22.1)	20.1 (18.0–22.0)	12.2 (10.9–13.4)	12.1 (11.1–13.3)	20.2 (18.6–21.9)	20.2 (18.7–21.8)	24.9 (22.7–26.9)	25.4 (23.3–27.3)	19.2 (17.2–21.0)	19.2 (17.5–21.1)
Dihomo-γ-linolenic acid (20:3n-6)	2.88 (2.51–3.33)	2.96 (2.55–3.41)	4.04 (3.43–4.59)	4.06 (3.48–4.72)	3.42 (2.97–3.97)	3.47 (3.00–4.01)	–	–	3.00 (2.64–3.41)	3.07 (2.65–3.51)	1.40 (1.24–1.61)	1.36 (1.21–1.53)	2.98 (2.54–3.46)	2.98 (2.55–3.46)
Arachidonic acid (20:4n-6)	10.6 (9.4–11.9)	10.6 (9.5–11.8)	9.3 (8.3–10.5)	9.3 (8.1–10.7)	10.4 (9.1–11.7)	10.3 (9.0–11.5)	8.6 (7.9–9.4)	8.7 (8.0–9.4)	10.9 (9.7–12.3)	11.0 (9.8–12.2)	10.2 (9.0–11.2)	10.1 (9.0–11.2)	11.2 (9.9–12.9)	11.4 (10.0–12.8)

* Results are reported as the median (interquartile range).

† The numbers of case patients and control patients are the maximum numbers for whom fatty acid measurements were available, and numbers varied by fatty acid.

‡ No value is given for these fatty acids, because they are present in negligible amounts in erythrocyte membranes.

**Figure 1.**
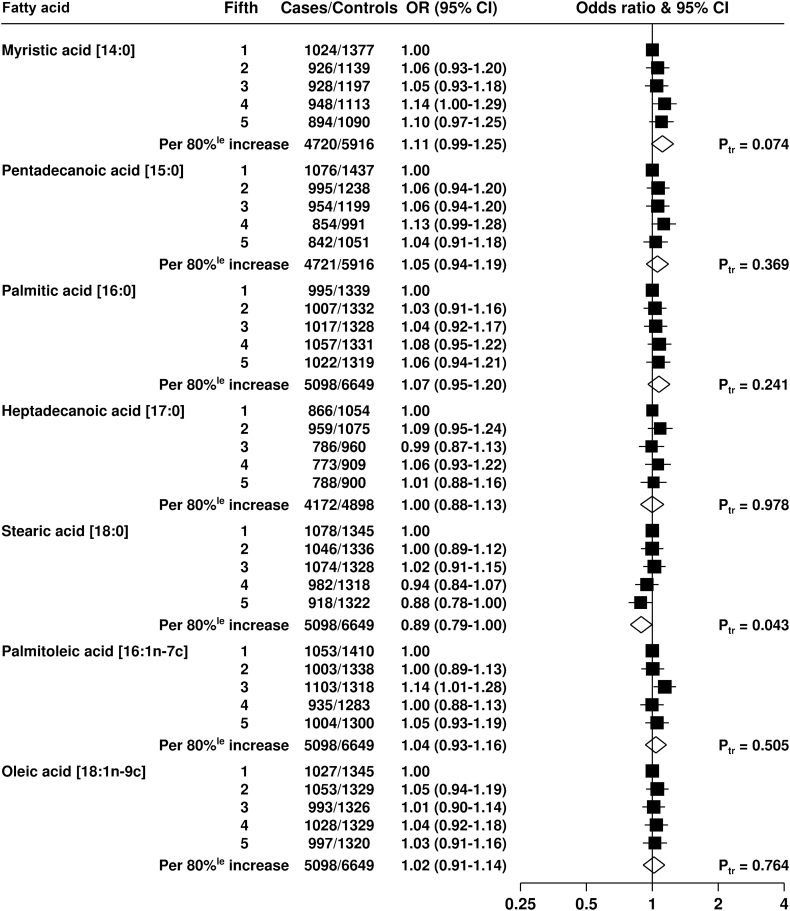
Odds ratios and 95% CIs for prostate cancer by study-specific fifths of circulating saturated and monounsaturated fatty acids. The odds ratios were conditioned on the matching variables and adjusted for age, marital status, education level, cigarette smoking, height, and BMI. The *P*_trend_ was calculated by replacing the fifths of the fatty acid with a continuous variable that was scored as 0, 0.25, 0.5, 0.75, and 1 in the conditional logistic regression model. All statistical tests were two-sided. 80%^le^ = 80 percentile; CI = confidence interval; P_tr_ = *P*_trend_.

For the saturated and monounsaturated fatty acids (Figure 1), there were no associations of the proportions of myristic (14:0), pentadecanoic (15:0), palmitic acid (16:0), heptadecanoic acid (17:0), palmitoleic acid (16:1n-7), or oleic acid with risk of total prostate cancer. However, stearic acid (18:0) was inversely related to total prostate cancer risk; compared with men in the lowest fifth, men in the highest fifth had a 12% lower risk (OR = 0.88, 95% CI = 0.78 to 1.00, *P*_trend_ = .043).

**Figure 2.**
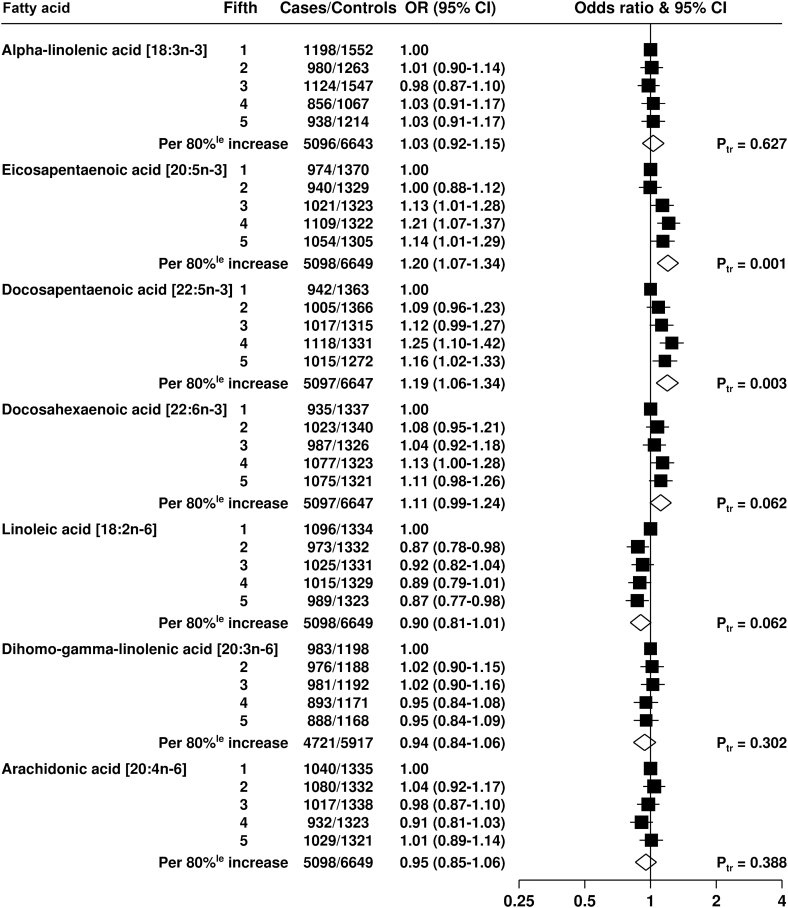
Odds ratios and 95% CIs for prostate cancer by study-specific fifths of circulating polyunsaturated fatty acids. The odds ratios were conditioned on the matching variables and adjusted for age, marital status, education level, cigarette smoking, height, and BMI. The *P*_trend_ was calculated by replacing the fifths of the fatty acid with a continuous variable that was scored as 0, 0.25, 0.5, 0.75, and 1 in the conditional logistic regression model. All statistical tests were two-sided. 80%^le^ = 80 percentile; CI = confidence interval; P_tr_ = *P*_trend_.

For the polyunsaturated fatty acids (Figure 2), there was no statistical association between alpha-linolenic acid (18:3n-3) composition and total prostate cancer risk; the odds ratio for the highest fifth compared with the lowest fifth was 1.03 (95% CI = 0.91 to 1.17). Eicosapentaenoic acid was positively related to risk of total prostate cancer (OR for the highest compared with the lowest fifth = 1.14, 95% CI = 1.01 to 1.29, *P*_trend_ = .001), as was n-3 docosapentaenoic acid (22:5n-3) (OR for the highest compared with the lowest fifth = 1.16, 95% CI = 1.02 to 1.33, *P*_trend_ = .003), but docosahexaenoic acid was not statistically associated with risk. There were no statistical associations between the proportions of the three major n-6 polyunsaturated fatty acids, linoleic, dihomo-gamma-linolenic (20:3n-6), and arachidonic, and total prostate cancer risk.

Results for groups of fatty acids, study-specific and subgroup analyses are shown in Supplementary Figures 1–30 (available online). There was a statistically significant inverse association between total n-6 polyunsaturated fatty acids and prostate cancer (*P*_trend_ = .022) but no statistical association with the long-chain n-6 fatty acids (*P*_trend_ = .472) (Supplementary Figure 1, available online). The odds ratios for the highest vs the lowest fifth of total and long-chain n-3 polyunsaturated fatty acids were 1.16 (95% CI = 1.02 to 1.31, *P*_trend_ =0.013) and 1.17 (95% CI = 1.03 to 1.32, *P*_trend_ = .011), respectively. There were statistically significant inverse associations of both n-6 to n-3 ratios with prostate cancer risk; the odds ratios for the highest vs the lowest fifth of the total and long-chain n-6 to n-3 ratios were 0.85 (95% CI = 0.74 to 0.96, *P*_trend_ = .005) and 0.78 (95% CI = 0.69 to 0.89, *P*_trend_ = .003), respectively.

There was evidence for heterogeneity between studies for eicosapentaenoic acid and docosapentaenoic acid with risk of total prostate cancer (*P* = .022 and <.001, respectively) (Supplementary Figures 10 and 11, available online). There was also evidence for heterogeneity between studies in the associations of palmitoleic acid and prostate cancer risk (*P* = .039) (Supplementary Figure 7, available online), and dihomo-gamma-linolenic acid and prostate cancer (*P* = .015) (Supplementary Figure 14, available online).

The associations of stearic acid did not differ by age at diagnosis, years from blood collection to diagnosis, year of diagnosis, stage of disease, presence of aggressive disease, grade of disease, age at blood draw, PSA concentration at blood draw, university education, BMI, smoking status, alcohol consumption, or family history of prostate cancer (*P*_heterogeneity_ > .05) (Supplementary Figure 20, available online). Eicosapentaenoic acid was statistically significantly positively associated with prostate cancer risk in men without a university education but not in more educated men (*P*_heterogeneity_ = .003), among men aged 60 years and over at blood draw but not in younger men (*P*_heterogeneity_ = .024), and among men without a family history of prostate cancer but not in men with a history of prostate cancer (*P*_heterogeneity_ = .044) (Supplementary Figure 24, available online). Docosapentaenoic acid was statistically significantly positively associated with prostate cancer risk in men diagnosed from year 2000 onwards but not among men diagnosed prior to this (*P*_heterogeneity_ < .001), among men with low grade disease (OR for an 80 percentile increase = 1.24, 95% CI = 1.10 to 1.41) but not in men with high grade disease (OR = 0.68, 95% CI = 0.45 to 1.03, *P*_heterogeneity_ = .006), and among men aged 60 years and over at blood draw but not in younger men (*P*_heterogeneity_ = .047) (Supplementary Figure 25, available online). The subgroup results for the other fatty acids are in Supplementary Figures 16–19, 21–23, and 26–29 (available online). Of the 143 tests for heterogeneity presented in these figures, there was statistically significant heterogeneity for myristic acid and age at diagnosis (Supplementary Figure 16, available online), palmitoleic acid and grade of disease (Supplementary Figure 21, available online), alpha-linolenic acid and both stage of prostate cancer and alcohol consumption (Supplementary Figure 23, available online), and arachidonic acid and both aggressive disease and cigarette smoking (Supplementary Figure 29, available online).In further analyses of the 14 fatty acids in relation to the risk for aggressive prostate cancer (Supplementary Figure 30, A and B, available online), the only association found was that the risk of aggressive disease was lower in men with the highest proportion of arachidonic acid (OR highest vs the lowest fifth = 0.70, 95% CI = 0.45 to 1.10, *P*_trend_ = .040).

## Discussion

The main findings from this collaboration were that men with lower concentrations of stearic acid and higher concentrations of eicosapentaenoic and docosapentaenoic acid had a modestly (approximately 15%) greater risk of developing prostate cancer, which did not differ by stage or aggressiveness of disease. This collaboration has brought together and reanalyzed data from seven prospective studies, representing almost 95% of the worldwide data on the association between circulating fatty acids and prostate cancer risk. While data from the Janus study of 141 case patients (6) and the Alpha-Tocopherol, Beta-Carotene Cancer Prevention Study (ATBC) of 198 case patients (7) were not available for this analysis, their results do not differ materially from those reported here, and it is unlikely that these data would change the associations described.

A higher proportion of stearic acid was associated with a lower risk of total prostate cancer. While short-term studies have shown that an increased consumption of foods rich in stearic acid increases the proportion of these fatty acids in the circulation (19–21), such relations have not been seen in some studies of long-term intake in free-living populations (22), suggesting that the level of this fatty acid in blood does not provide a simple index of intake (23). Besides dietary intake, lower levels of stearic acid in the blood could be a result of altered metabolism of this fatty acid (24), although evidence for these pathways being implicated in the development of prostate cancer is limited. While we did not observe statistically significant heterogeneity in the association of stearic acid with risk of prostate cancer according to the number of years between blood collection and diagnosis, some reverse causation bias is still possible, because the latency period of prostate cancer might be up to 10 years (25).

Both eicosapentaenoic and docosapentaenoic acid were weakly positively associated with prostate cancer risk; however, there was heterogeneity between studies. There were statistically significant positive associations in EPIC and SELECT for eicosapentaenoic acid, and in PCPT and SELECT for docosapentaenoic acid but statistically significant inverse associations for eicosapentaenoic and docosapentaenoic acid in the PHS. There were differences between the studies in the types of blood samples used to measure fatty acids; the PHS was the only study to measure the fatty acid composition of whole blood; however, we accounted for this difference by dividing participants into study-specific fifths (18). It is possible that differences between studies in the underlying participant population (eg, men participating in a randomized trial vs an observational study) might account for some of this heterogeneity. It is therefore difficult to draw any conclusions about eicosapentaenoic and docosapentaenoic acid from this analysis. A possible explanation for the positive associations between eicosapentaenoic and docosapentaenoic acid and total prostate cancer risk might involve the behavior of health-conscious men who may have both a high consumption of fish and a relatively high likelihood of having a PSA test in countries where the overall rate of PSA testing is lower than in the United States (26–28). This has been previously described in the PHS (29). Although the positive associations of circulating eicosapentaenoic acid and docosapentaenoic acid with prostate cancer risk did not differ statistically significantly according to whether the prostate cancer was diagnosed before or after the introduction of PSA testing in the early 1990s, the risk was greater in post-2000 cancers than in earlier cancers, and the study that showed statistically inverse associations of eicosapentaenoic and docosapentaenoic acid with prostate cancer risk (PHS) had a large proportion of cases diagnosed before 1995, suggesting that these discrepant associations might be partly explained by detection bias. Further support for this idea comes from the findings of statistically positive associations of docosapentaenoic acid with localized, nonaggressive and low-grade disease but not advanced, aggressive, or high-grade prostate cancer.

The results from this study do not support the hypothesis that a higher proportion of alpha-linolenic acid increases the risk of prostate cancer, as reported in an earlier analysis of the PHS (30). A recent meta-analysis of published results from 16 studies reported an overall positive association between alpha-linolenic acid and prostate cancer risk (31), but this was based on both retrospective and prospective studies and studies of both dietary intake and tissue levels. Our results, showing little or no association, represent the most up-to-date and comprehensive summary of the prospective association between circulating alpha-linolenic acid and prostate cancer risk.

For the other fatty acids examined, there was no evidence for associations with risk for total prostate cancer. The supplementary analyses of the sums of n-6 and n-3 fatty acids and their ratios showed some statistically significant associations, but these results are largely predictable from the results for the individual fatty acids and are difficult to interpret. Several analyses described in the Supplementary figures (available online) showed heterogeneity between studies or subgroups, but these results are difficult to interpret because of the large number of comparisons made, making it difficult to rule out the play of chance. However, the finding of a statistically inverse association between arachidonic acid and aggressive prostate cancer is of potential interest. While there have been many experimental studies examining the putative effects of arachidonic acid on promoting prostate cancer initiation and progression (32), there is almost no evidence to support mechanisms whereby arachidonic acid inhibits prostate cancer progression.

These results represent the associations of prostate cancer risk with fatty acid measures from a single blood sample for each man, taken on average five years before cancer diagnosis in the cases. The extent to which a single blood sample reflects long-term values has not been studied extensively, but a study of serum phospholipid fatty acids in blood samples taken three to four years apart reported correlation coefficients of 0.7 for stearic acid and 0.5 for eicosapentaenoic acid (33). This might mean that the associations between the “usual” proportion of circulating fatty acids and total prostate cancer risk are likely to be stronger than those reported here.

In summary, the results from this collaborative analysis of over 5000 cases and 6000 controls showed that men with lower circulating stearic acid and higher eicosapentaenoic and docosapentaenoic acid had a slightly elevated risk of developing total prostate cancer. However, it is not clear whether these modest associations indicate any causal relationships between fatty acids and prostate cancer risk.

## Funding

Centralized pooling, checking, and analysis of data were supported by Cancer Research UK (grant number C570/A11691). We thank the men who participated in the collaborating studies, the research staff, the collaborating laboratories, and the funding agencies in each of the studies. The Carotene and Retinol Efficacy Trial was supported by the National Cancer Institute at the National Institutes of Health (grants R01CA96789, U01CA63673, and N01PC35142). The Physicians’ Health Study was supported by the US Department of Defence (grant W81XWH-11-1-0529) and the National Institutes of Health (grants CA42182, CA58684, CA90598, CA141298 CA97193, CA34944, CA40360, CA131945, P50CA90381, 1U54CA155626-01, P30DK046200, HL26490, and HL34595). SWOG’s contribution of data from the Prostate Cancer Prevention Trial and the Selenium and Vitamin E Cancer Prevention Trial was supported by a NCI/DCP grant (CA37429).

## Notes

The authors are solely responsible for the study design, data collection, analysis and interpretation of the data, writing the article, and the decision to submit the article for publication. All authors contributed to these analyses and read and approved the final manuscript. The sponsors had no role in the study’s design, data collection, analyses, interpretation of results, preparation of the manuscript, or the decision to submit the manuscript for publication. The authors have no conflicts of interest to declare.
